# Generation and Validation of a *Shewanella oneidensis* MR-1 Clone Set for Protein Expression and Phage Display

**DOI:** 10.1371/journal.pone.0002983

**Published:** 2008-08-20

**Authors:** Haichun Gao, Donna Pattison, Tingfen Yan, Dawn M. Klingeman, Xiaohu Wang, Joseph Petrosino, Lisa Hemphill, Xiufeng Wan, Adam B. Leaphart, George M. Weinstock, Timothy Palzkill, Jizhong Zhou

**Affiliations:** 1 Institute for Environmental Genomics, University of Oklahoma, Norman, Oklahoma, United States of America; 2 Environmental Sciences Division, Oak Ridge National Laboratory, Oak Ridge, Tennessee, United States of America; 3 Baylor College of Medicine, Houston, Texas, United States of America; Centre for DNA Fingerprinting and Diagnostics, India

## Abstract

A comprehensive gene collection for *S. oneidensis* was constructed using the lambda recombinase (Gateway) cloning system. A total of 3584 individual ORFs (85%) have been successfully cloned into the entry plasmids. To validate the use of the clone set, three sets of ORFs were examined within three different destination vectors constructed in this study. Success rates for heterologous protein expression of *S. oneidensis* His- or His/GST- tagged proteins in *E. coli* were approximately 70%. The ArcA and NarP transcription factor proteins were tested in an *in vitro* binding assay to demonstrate that functional proteins can be successfully produced using the clone set. Further functional validation of the clone set was obtained from phage display experiments in which a phage encoding thioredoxin was successfully isolated from a pool of 80 different clones after three rounds of biopanning using immobilized anti-thioredoxin antibody as a target. This clone set complements existing genomic (*e.g.*, whole-genome microarray) and other proteomic tools (*e.g.*, mass spectrometry-based proteomic analysis), and facilitates a wide variety of integrated studies, including protein expression, purification, and functional analyses of proteins both *in vivo* and *in vitro*.

## Introduction

*Shewanella oneidensis* MR-1, a facultatively anaerobic γ-proteobacterium, is capable of utilizing a diversity of organic compounds and metals to obtain energy needed for growth and survival. Since the genome sequence of *S. oneidensis* MR-1 was released in 2002 [1], many biological processes in *S. oneidensis* MR-1 have been investigated in a high-throughput manner by utilizing methods such as whole-genome microarrays, liquid chromatography-mass spectrometry (LC/MS), gas-chromatography-mass spectrometry (GC/MS), and nuclear magnetic resonance (NMR). These studies have greatly improved our understanding of lipopolysaccharides [2], small proteins [3], protein complexes [4] and metabolic flux [5]. In addition, new information has been obtained on regulatory responses to environmental stresses [6]–[12], heavy metals [13]–[16], and to an array of electron acceptors [17]. However, the resources necessary to support systematic *in vivo* and *in vitro* approaches for the analysis of protein function as well as protein-protein and DNA-protein interactions are still limited for *S. oneidensis*.

A comprehensive collection of individual open reading frames (ORFs) would be a powerful resource for *S. oneidensis* given its importance as a model organism for genetic and genomic studies of metal-reducing bacteria. A collection of such clones should be flexible and amenable for many *in vivo* and *in vitro* experimental systems as discussed in recent reports and reviews [18]–[22]. This resource would facilitate research in various aspects of functional genomics and proteomics in *S. oneidensis*, including but not limited to deciphering the functional activities of proteins, especially those involved in metal reduction [23]–[25], and building regulatory networks and interaction maps [26]–[27].

We describe here the generation and validation of a *S. oneidensis* clone set constructed using lambda recombinase (Gateway) technology [28]. The method circumvents restriction enzyme digestion and ligation of nucleic acid used in conventional cloning. By exploiting site-specific recombination, it provides a high-throughput means of cloning and subcloning ORF candidates of interest. The system involves cloning ORFs as PCR products into an entry plasmid that serves as the base for a set of clones. The entry vectors do not provide a means for protein expression or functional assays; they are a place-holder for the ORF. The ORF can then be transferred by site-specific recombination to a destination vector of choice. The conversion of the ORF to a destination vector allows for protein expression or functional studies in an organism of interest. With a wide variety of destination vectors, ORFs cloned into the entry vectors can be rapidly transferred for different applications.

## Results

### Updates in annotation of *S. oneidensis* genome

To create a comprehensive set of cloned genes for *S. oneidensis* the most important prerequisite is genome sequence information and the annotation for the complete set of predicted ORFs. The annotation of protein-coding genes in the *S. oneidensis* genome has changed over time. The genome was sequenced by TIGR (www.tigr.org) and the results were deposited in NCBI GenBank (AE014299 and AE014300) in 2002. *S. oneidensis* possesses a circular chromosome of 4,969,803 base pairs (bp) and a plasmid of 161,613 bp [1]. According to Heidelberg *et al.*, there are a total of 4,758 and 173 predicted protein-encoding open reading frames on the chromosome and plasmid, respectively [1]. The final released version of annotation at www.tigr.org by TIGR, however, contains a total of 4,938 protein coding genes. After deposition, the genome was reannotated by NCBI and this version (chromosome & plasmid) contains only 4,467 protein coding genes (www.ncbi.nlm.nih.gov) [29]. The genome was annotated again by the *Shewanella* Federation [30], which further reduced the number of protein coding genes to 4,192. Moreover, the latest annotation also revealed frameshift sequencing errors in 105 ORFs. These errors resulted in a number of changes including truncated, merged, and split genes as well as genes with altered start codons.

### Primer design, ORF amplification, and Cloning

The lambda recombination cloning method (Gateway) utilizes a DNA fragment flanked by *att* recombination sites that can be combined *in vitro* with an entry vector that also contains recombination sites and incubated with integration proteins to transfer the DNA fragment into the vector [28]. Consequently, the *att*B1 and *att*B2 sequences must be added during the PCR amplification at the 5′ end of each ORF-specific forward and reverse primer, respectively. The forward ORF-specific primers begin with the start codon except for genes containing a signal peptide sequence, with which the primers begin at the first codon after the signal peptide. This design prevents removal of any N-terminal fusion peptides in the destination vectors by host-cell signal peptidases. The reverse ORF-specific primers begin with the nucleotide before the stop codon in order to allow C-terminal fusion proteins to be expressed from destination vectors. Because of this design, the amino acids encoded in the final flanking *att* sequences are fused to each ORF-encoded sequence at the N- and C-termini of the proteins.

For the generation of the *S. oneidensis* clone set, 4,526 unique pairs of primers were initially designed based on the TIGR annotation. The changes in genome annotation, however, made a number of these primer pairs irrelevant with the end result and 4,030 primer pairs were valid for PCR amplification. All PCR amplification was performed in 96-well plates. From the first round of attempted amplification, a band corresponding to the specific ORF was obtained from 3504 ORFs. The second and third rounds produced 154 and 105 qualified PCR products, respectively. In total, 3,763 ORFs were generated with valid primer pairs by amplification, indicating a 93.3% success rate.

The recombination reactions used to insert the amplified PCR products into pDONR221 were performed in 96-well plates and subsequently were used to transform chemically competent DH5α *E. coli* cells. Initially, three colonies on LB plates containing kanamycin were picked and examined by PCR with M13 forward and reverse primers. Colonies were regarded as positive clones and stored unless a discrepancy was observed among three PCR products. For failed ORFs, more colonies were examined until three colonies exhibiting a correct size of PCR products were obtained. In total, 3,584 out of 3,763 amplified products were successfully captured in the pDONR221 entry vectors indicating a success rate of 95.4%. This represents approximately 85% of the 4,192 ORFs in *S. oneidensis* MR-1 genome according to the latest annotation. It is worth noting that the average size of ORFs failed in the recombination reaction was 2315 bp, nearly three times as large as the average size (845 bp) of all ORFs in the genome, implicating that the recombination reaction is less efficient for large genes.

### DNA sequence validation of cloned genes

DNA sequence analysis was performed on a collection of the clones to examine the levels of mutations associated with PCR and the cloning procedure. DNA sequencing was attempted for each entry clone from both the 5′ and 3′ directions and at least one additional attempt was made if the quality of the first reads for a particular clone was poor. DNA sequence data were obtained for at least one direction for 85.7% (3,070 of 3,584) of the genes and junction regions in the *S. oneidensis* pDONR221 entry clone collection. A subset of sequences were analyzed to determine the frequency and source of errors among the cloned genes. A total of 107,071 of 107,083 (99.99%) of the insert bases sequenced were correct in the 159 clones examined. It was not determined whether the incorrect bases were errors that exist in the original genome sequence or introduced during the cloning process. This error frequency is of the same magnitude observed for bacterial whole genome sequencing efforts (typically approximated at 1 error in 10,000 bases). The 12 errors affect nine of the 159 clones examined. The clone for SO3896 contained two frameshifts of one and three nucleotides in length (accounting for four of the errors). The remaining eight errors, two frameshifts and six substitutions, were found in eight genes. While five of the six substitutions led to nonconservative amino acid changes (Met to Ala, Gly to Arg (twice), Ala to Val, and Ser to Pro), one resulted in a conservative substitution (Thr to Ser). Five clones, including SO3896, contained errors in the oligo-encoded 5′ or 3′ adapter *att* sequences that could affect the frequency of subsequently recombination into destination plasmids. In clones examined where the entire insert gene was sequenced, 31 of 32 clones were found to have no discrepancies with the published gene sequences. Therefore, the clone set contains relatively few mutations due to the PCR and cloning procedures.

### Expression and characterization of *S. oneidensis* two-component response regulatory proteins

The advantage of the lambda recombinational cloning system is that a single set of clones can be converted to many different functional destination vectors. The efficiency of protein expression from the *S. oneidensis* clones in destination vectors was examined with a set of two-component transcriptional regulatory proteins. In order to respond to changing environments, bacteria such as *S. oneidensis* utilize global regulatory mechanisms such as the two-component regulatory system to simultaneously mediate the transcription of numerous operons. Two-component systems are composed of a sensor protein that is usually in the membrane and binds to an environmental signaling molecule. In response to the signal, the sensor protein autophosphorylates at a histidine residue and then transfers this phosphoryl group to a response regulatory protein that binds DNA at operons that make up the regulon to activate or repress transcription [31].

**Figure 1:**
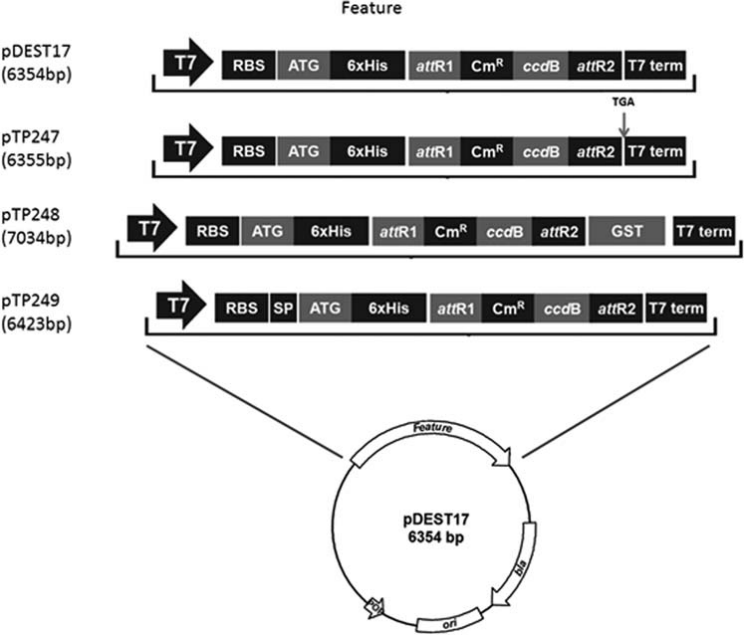
Destination vectors constructed in this study. A variety of destination vectors derived from pDEST17 are shown.

Based on the genome sequence annotation, thirty response regulatory proteins were selected and transferred from the pDONR221 plasmid to the pTP247 destination plasmid which is a modified version of the commercially available pDEST17 His-tag expression vector (Fig. 1). The stop codon of the destination vector pDEST17 is not in-frame with the upstream gene sequence when conversions are made using the pDONR221 vector resulting in the addition of 22 extra amino acids to the end of the gene product. A new, in-frame, stop codon (TGA) was introduced by site directed mutagenesis to create the pTP247 plasmid which avoids the addition of the extra 22 amino acids. LR Clonase (Invitrogen) was used to catalyze homologous recombination between the donor and destination vectors thus creating an expression plasmid for the ORF of interest. The pTP247-ORF plasmids were introduced into *E. coli* cells and protein expression was induced with IPTG. Twenty-one out of 30 of the proteins tested were expressed in *E. coli* cells as evaluated by the presence of a band of the expected size after resolving the soluble and insoluble fractions of whole cell protein lysates by SDS-PAGE and Coomassie Blue staining (Table 1). Expression of four of the proteins tested led to poor cell growth possibly due to a toxic effect of the introduced protein. Five of the proteins were not expressed under the conditions tested. The response regulatory proteins that were expressed were found predominantly in insoluble inclusion bodies. Formation of inclusion bodies is common for heterologous protein expression in *E. coli* and recent studies suggest optimization of growth conditions can lead to increased soluble protein expression [32].

**Table 1:** Expression data for *S. oneidensis* response regulator proteins in *E. coli*

ORF	Predicted protein length (a.a)	Protein expression	Soluble or Inclusion body	Annotation
SO2538	370	Yes	Inc. body	Response regulator
SO4718	460	No^a^		Sigma-54 dependent Response regulator
SO2426	237	No^a^		DNA-binding Response regulator
SO0622	224	No^a^		DNA-binding Response regulator
SO0351	198	Yes	Inc. body	DNA-binding Response regulator
SO2193	230	Yes	Inc. body	DNA-binding Response regulator
SO2104	219	Yes	Inc. body	DNA-binding Response regulator
SO2540	192	No^a^		Response regulator
SO2125	208	Yes	Inc. body	chemotaxis protein CheD
SO1860	214	Yes	Inc. body	DNA-binding Response regulator
SO1946	224	Yes	Inc. body	Transcriptional regulatory protein, PhoP
SO4647	231	Yes	Inc. body	DNA-binding Response regulator
SO0860	339	Yes	Inc. body	Response regulator
SO2539	658	Yes	Inc. body	Response regulator
SO2366	525	Yes	Inc. body	Response regulator
SO1416	199	Yes	Inc. body	DNA-binding Response regulator
SO3594	237	Yes	Inc. body	Transcriptional regulatory protein, RstA, putative
SO0545	575	No		Response regulator
SO2541	441	Yes	Inc. body	Response regulator
SO2547	121	No		Response regulator
SO0059	229	Yes	Inc. body	Transcriptional regulatory protein, KdpE
SO1558	239	Yes	Inc. body	Phosphate regulon Response regulator, PhoB
SO3982	209	Yes	Soluble	DNA-biding nitrate/nitrite Response regulator
SO4003	357	Yes	Soluble	Response regulator
SO4172	183	No		DNA-binding Response regulator
SO4388	227	Yes	Inc. body	DNA-binding Response regulator
SO4477	228	Yes	Inc. body	Transcriptional regulatory protein, CpxR
SO4633	241	No		Transcriptional regulatory protein, OmpR
SO4428	225	No		DNA-binding Response regulator
SO4487	220	Yes	Inc. body	DNA-binding Response regulator

a expression of the protein has a strong negative effect on growth of *E. coli* host cells

**Figure 2:**
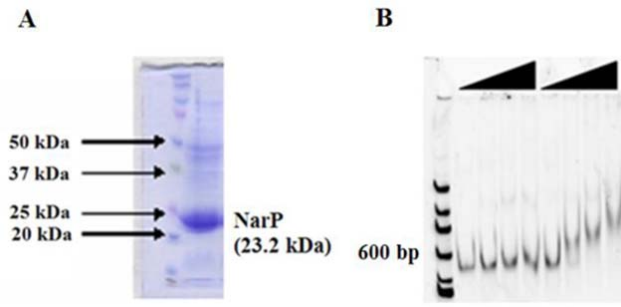
SDS-PAGE of *S. oneidensis* NarP expressed from the destination vector pTP247. A. Lane 1: protein marker; Lane 2: Soluble protein after lysis of *E. coli* expressing NarP protein. B. Electrophoretic gel shift assays of NarP and phosphorylated NarP-P. Increasing concentrations (0, 0.65, 1.3, 2.6, and 5 µM) of unphosphorylated NarP and phosphorylated NarP are shown. DNA is stained with CyberGreen. The 100bp ladder was used in the experiments.

Protein expression and purification studies were pursued further for two of the response regulatory proteins, ArcA and NarP. As shown previously by Gao et al. [33], the purified ArcA protein can bind specifically to promoter DNA when phosphorylated/activated using carbamoyl phosphate according to electrophoretic gel mobility shift assays [33]. The non-phosphorylated protein did not show any specific DNA binding activity [33]. SO3982, showing sequence homology to *E. coli* NarP (72% similarity), was named as NarP in the latest annotation. NarP is a response regulator belonging to the LuxR family. Evidence from work in *E. coli* suggests that NarP may regulate its own expression [34], as well as expression of NrfA, a formate-dependent nitrite reductase enzyme [35]. In *S. oneidensis,* NarP and NrfA share the same promoter region, although the genes are transcribed in opposite directions. This promoter region was amplified via PCR and used in electrophoretic mobility shift assays. The cloned *narP* gene in the pDONR221 vector was transferred to the pTP247 His-tag destination vector and the NarP protein was expressed and purified in soluble form using metal affinity chromatography (Fig. 2A). The purified protein was phosphorylated with carbamoyl phosphate and electrophoretic mobility shift (EMSA) DNA binding studies indicated the phosphorylated SO3982 protein bound to a PCR product containing *narP-nrfA* promoter DNA while nonphosphorylated SO3982 protein did not (Fig. 2B). These results indicate that NarP phosphorylation is required for DNA binding. These studies demonstrate that the clone set and the pTP247 destination plasmid can be used to successfully express and purify proteins in *E. coli*.

### Construction of additional destination vectors for protein expression

Two additional destination vectors that are useful for protein expression were constructed for this study (Fig. 1). The pTP249 plasmid is a destination vector for the expression of proteins in *E. coli* that are secreted to the periplasmic space. It was constructed by inserting a DNA fragment encoding the *E. coli pelB* secretion signal sequence into the pDEST17 plasmid. Upon conversion of genes from the pDONR221 plasmid to pTP249, the proteins are secreted to the periplasm of *E. coli*. This plasmid was constructed in order to express periplasmic proteins in their normal environment. This is particularly important for the expression of proteins containing disulfide bonds that will be oxidized in the periplasm but not the cytoplasm.

**Figure 3:**
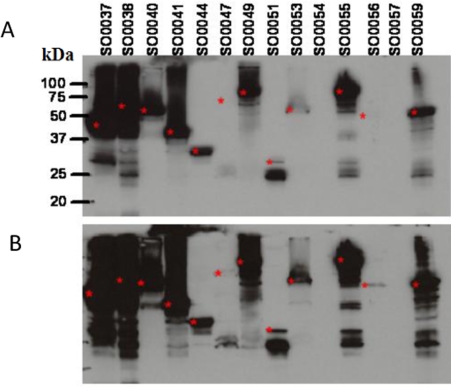
Expression of *S. oneidensis* ORFs from the pTP248 double-tag destination plasmid. A. The protein expression levels of ORFs were evaluated from whole cell protein lysates of *E. coli* by SDS-Page fractionation and immunodetection using anti-GST antibody. The *S. oneidensis* ORF number is shown above each lane. The red asterisks indicate the position of a band that is consistent with the predicted molecular weight of the fusion protein. Note that for ORFs SO0047 and SO0056, a band is not visible in panel A but is visible in the longer film exposure of panel B. The predicted molecular weights (kDa) of the fusion proteins are: SO0037, 40.7; SO0038, 65.1; SO0040, 61.2; SO0041, 41.4; SO0044, 39.7; SO0047, 75.1; SO0049, 86.9; SO0051, 34.3; SO0053, 66.8; SO0054, 74.2; SO0055, 94.7; SO0056, 59.3; SO0057, 79.9; SO0059, 57.1. B. Identical to A. but with a longer film exposure.

The pTP248 destination vector was constructed in order to express proteins with purification tags at both the N and C-termini of the protein. After conversion of genes from the clone set in pDONR221 to pTP248 and expression in *E. coli*, the proteins contain a 6x His-tag at their N-terminus and the GST protein fused to the C-terminus. A set of *S. oneidensis* genes in pDONR221 were converted to the pTP248 vector and protein expression was evaluated upon IPTG induction in *E. coli* to estimate the percentage of gene products that can be expressed from this construct. These genes, from the SO0037 to SO0059 ORF region of the chromosome, encode proteins with a range of different functions including metabolism, transcription regulation, transporters and conserved hypotheticals. As seen in Fig. 3, approximately 60–70% of the *S. oneidensis* gene products were expressed in *E. coli* and detected in whole cell lysates by Western blotting using an anti-GST antibody.

### Phage display of *Shewanella* ORFs

**Figure 4:**
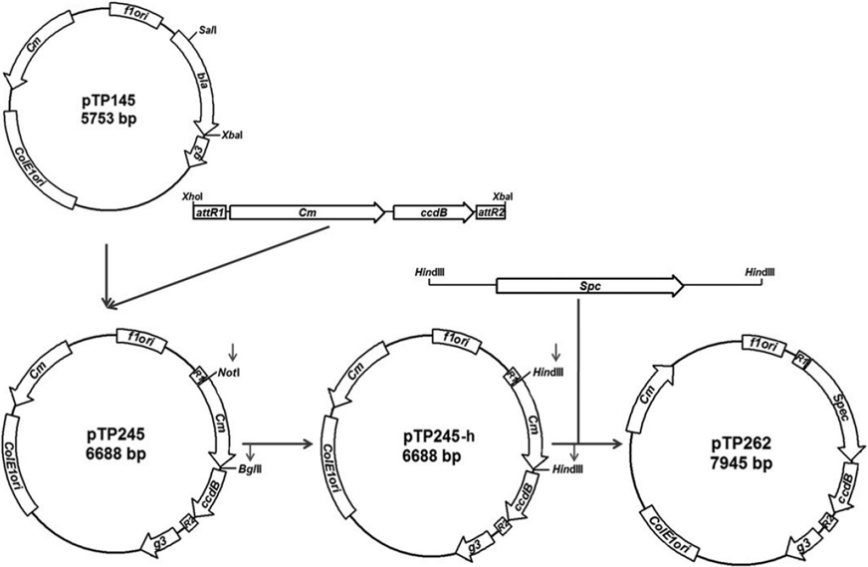
Phage display destination vector constructed in this study. M13 filamentous phage display vector pTP262 is a destination plasmid that can be used for insertion of ORFs from the pDONR clone set to create phage display plasmids.

Phage display is an approach for the study of interactions between proteins, proteins and ligands, and proteins and DNAs. Several different types of bacteriophages have been used as platforms for phage display but the most commonly used is M13 bacteriophage [36]. The phage display is one of the techniques chosen by the Shewanella Federation for functional analyses thus a great effort was made to create and validate a phage display system for the bacterium. A destination vector was made by inserting a cassette containing *att*R sites flanking a chlorampenicol resistance gene and the *ccd*B gene for counter selection, into the pTP145 phage display vector [37]. The pTP145 phage display plasmid allows display of the TEM-1 β-lactamase enzyme as a fusion to the M13 gene III capsid. The *att*R cassette was inserted between the β-lactamase signal sequence and gene III which, upon recombination conversion with a pDONR221 vector would place a *S. oneidensis* ORF in position to be displayed on the surface of M13 bacteriophage (Fig. 4). This destination vector was named pTP262.

In order to test the usefulness of the bacteriophage M13 phage display system implemented with pTP262, a sub-library was constructed and tested for the ability to isolate a gene by biopanning. The phage display system was tested using a rabbit polyclonal antibody to the *E. coli* thioredoxin protein (Materials and Methods). The *S. oneidensis* thioredoxin protein is 76% identical to the *E. coli* protein and it was reasoned that the polyclonal antibody against the *E. coli* protein would also bind the *S. oneidensis* protein. The library to be used for phage display consisted of 80 clones from the SO0337 to SO0516 region of the genome which includes SO0406 encoding thioredoxin (*trx*A). Plasmid DNA from each pDONR221 clone was prepared and the set of 80 plasmids was pooled. The pooled plasmids were then converted from the pDONR221 vector to the pTP262 destination vector in batch using LR Clonase recombination enzyme [28]. The reaction products were introduced into *E. coli* by electroporation and the transformed cells were spread on agar plates containing chloramphenicol. Six colonies were picked and analyzed by restriction enzyme digestion to ensure that the conversion reaction was efficient and the library contained inserts into the pTP262 plasmid. Five of six clones contained inserts and the five inserts were of different size indicating the reaction of efficient and the library is diverse. Approximately 10,500 colonies on the agar plates were pooled to create the sub-library of *S. oneidensis* genes in pTP262.

**Figure 5:**
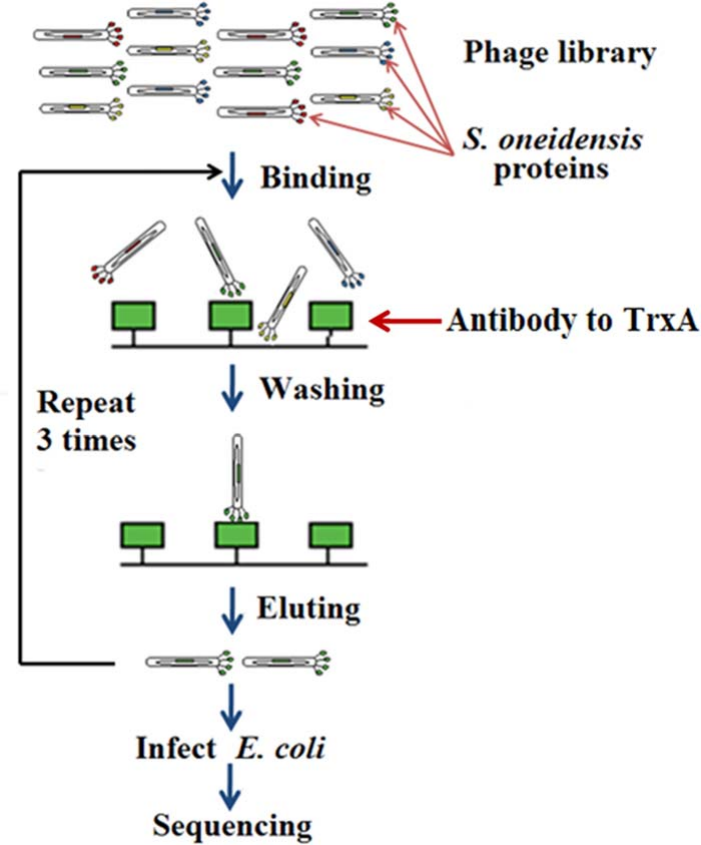
*In vitro* biopanning protocol. Briefly, phages were incubated with immobilized anti-*E. coli* thioredoxin antibody, non-binders were removed by washing, bound phage were eluted, amplified, and the process was repeated. Eluted phage clones from round 3 were DNA sequenced.

Phage were prepared from the library using helper phage as previously described [37]. Three rounds of biopanning were performed whereby a polyclonal antibody against the highly similar *E. coli* thioredoxin was immobilized and the phage library was allowed to bind, unbound phage were washed away and bound phage were eluted and amplified for the next round of panning (Fig. 5). After the third round of biopanning, the eluted phages were used to infect *E. coli*. Six clones were chosen from the round 3 population and the identity of the gene for each of the six clones was determined by DNA sequencing to be *trxA*, which encodes thioredoxin. This result indicates the *trxA* phage clone was enriched during the panning process and dominated the population after three rounds. Therefore, it was concluded that the thioredoxin was efficiently displayed on the surface of the phage after expression from the pTP262 destination vector. These results suggest that pooling sets of ORF clones is an efficient means of converting the clone set to a phage display vector and that antibody-antigen interactions can be enriched by phage display.

## Discussion

While molecular cloning with restriction enzymes has been a well-established procedure for more than three decades, the emergence of the Gateway (Invitrogen), the Creator (Clontech), the Univector [38], and the MAGIC [39] cloning systems makes the creation of an ORF repositories feasible. Such site-specific recombination based cloning systems have been successfully employed for building a number of clone sets [18], [20]–[22], [40]–[42]. In recent years, the lambda recombinase (Gateway) system has been become popular because of its high-fidelity and cost efficient properties [18], [20], [22], [41]–[42]. In this study, an ORF clone set for *S. oneidensis* was created using the lambda recombinase system [28]. ORFs within entry vectors in this system can be readily transferred into multiple destination vectors, making the clone set a useful resource for research groups studying this microorganism, especially those expressing proteins *in vivo* or *in vitro* and using a variety of experimental analyses [26], [43]–[46].

Due to the acquisition of new experimental data and advanced sequence analysis methods, annotation of protein-coding genes in the *S. oneidensis* genome has become increasingly accurate. The clone set was constructed using primer pairs based on the most recent annotation. As outlined above, 3,584 ORFs were captured in the pDONR221entry vector, with a success rate of 93.3 % for PCR amplification and 95.4% for cloning. These rates are in agreement with those observed from the work on *Brucella melitensis* [41]. Despite multiple attempts at PCR amplification and cloning, a number of ORFs were not amplified and/or captured in the donor vector. It appears that the ORF size affects the BP cloning efficiency negatively. In average, the ORFs failed to be captured in the BP reaction were nearly three times larger than the ORFs in the genome. The *S. oneidensis* clone set was validated by sequencing a total of 107,083 insert bases. 12 mutations were identified, corresponding to one error per 8924 bp or a mutation rate of 0.01%. This is significantly lower than those observed in the other clone sets [18], [40]. There are multiple factors that could contribute to mutations. The mutation rate is 1 error every 2673 bp in the *Treponema pallidum* clone set, which employed Advan*Taq* DNA polymerase (Clontech) for PCR amplification [40]. The higher fidelity of *Pfu* turbo DNA polymerase used in this study likely accounts for the difference in mutation rates. On the other hand, a mutation rate of 1 error every 450 bp obtained in the *Pseudomonas aeruginosa* clone set was suggested by authors to be largely due to the high-GC-content ORFs [18]. Strikingly, PCR amplification during construction of this *P. aeruginosa* clone set yielded a success rate of 99%. *Taq* polymerase is reported to have 4-10 times higher processitivity than *Pfu* turbo polymerase although the enzymes differ little in amplification efficiency [47]. Nonetheless, it is important to amplify ORFs for a clone set with an enzyme of high amplification efficiency that also has a low error rate.

Although the Gateway system offers multiple destination vectors for a variety of analyses, we developed a series of destination plasmids to better suit the needs of this study. The backbone of these plasmids is pDEST17, one of the commercial destination vectors specifically designed for the Gateway system (Invitrogen). With introduction of a new, in-frame, stop codon, the resulting His-tag expression vector pTP247 functions the same as pDEST17 without the addition of the extra 22 amino acids to the end of the gene product. Other plasmids were designed for expression of proteins with a His-GST double-tag (pTP248) and for secreted or periplasmic proteins (pTP249) as well as for M13 phage display (pTP262). Except for pTP249, all these plasmids were used to validate selected clones from the collection. The newly constructed destination plasmids are suitable for use with other clone sets or with individual genes.

To establish that the *S. oneidensis* clone set could be used for protein expression and functional studies, three sets of ORFs were examined for expression of His-tag proteins, expression of His/GST-tag proteins, or for effective display on phage. A total of 21 out of 30 (70%) predicted two-component transcriptional regulators from *S. oneidensis* were successfully expressed in the His-tag format. While the reason for failure in expression of five proteins is unknown, expression of four genes was toxic to *E. coli* growth. In-depth studies with both ArcA and NarP revealed that purified proteins were suitable for *in vitro* binding assays, further validating the use the clone set for protein expression [33]. A similar success rate was obtained for expression of 14 His-GST double tagged proteins examined by Western blotting using an anti-GST antibody.

The use of the *S. oneidensis* clone set for functional studies was tested using a phage display system. Phage display is a useful method for studying protein-ligand interactions [36]. The method involves the fusion of peptides or proteins to a coat protein of a bacteriophage. This results in display of the fused protein on the exterior of the phage, while the DNA encoding the fusion resides within the virion. The physical linkage between the displayed protein and the DNA encoding it allows screening of vast numbers of proteins for ligand-binding properties [36]. With this technology, a phage clone encoding thioredoxin TrxA was isolated from a sub-library consisting of 80 clones.

It is evident that the *S. oneidensis* clone set can be used for expression of functional *S. oneidensis* proteins in *E. coli* using the appropriate destination vectors. Destination vectors are available for a number of protein expression formats as well as for functional studies such as phage display and yeast two-hybrid assays. Note that it should be possible to construct a destination vector that can be used to expression proteins directly in *S. oneidensis*. In combination with whole-genome microarrays and proteomics databases [3], [6], the clone set is a useful resource for integrated studies at the whole genome level. The functionality of many genes cannot be determined from sequence similarity alone. Studies on *S. oneidensis* have revealed many surprising findings about proteins that have been well-studied in other model organisms such as *E. coli*. These include the observation that reduction pathways for all electron acceptors but TMAO share a pivot electron transfer protein CymA [48], that an Arc system without ArcB mediates aerobic growth [33], [49], that the *E. coli* Fnr analog has no significant role in anaerobiosis [50], and that Crp functions under anaerobic but not aerobic conditions [51], to name a few. In this respect, the clone set will be particularly useful because it facilitates protein expression, purification, and a variety of functional analyses

## Methods

### Bacterial strains, plasmids, primers, and culture conditions

A list of *Escherichia coli* strains, plasmids, and primers used in this study is given in Table 2. The cloning to obtain master clones, protein expression, and the Gateway destination cloning were performed using *E. coli* strains DH5α, BL21(DE3)*, and DB3.1, respectively. Other recombinant DNA experiments were performed using *E. coli* XL1-Blue (Stratagen). Unless otherwise noted, *E. coli* strains and *S. oneidensis* MR-1 were grown in Luria-Bertani (LB, Difco) medium at 37°C and room temperature for genetic manipulation, respectively. When needed, the growth medium was supplemented with antibiotics at the following concentrations: ampicillin at 50 µg/ml, kanamycin at 50 µg/ml, chloramphenicol at 12.5 µg/ml, and spectinomycin at 50 µg/ml.

**Table 2:** Strains, plasmids, and primers used in this study

Strain or plasmid	Characteristic or relevant feature	Reference or source
*E. coli*		
DH5α	F- φ80*lac*ZΔM15 Δ(*lac*ZYA-*arg*F) U169 *rec*A1 *end*A1 *hsd*R17 (r_k_−, m_k_+) *pho*A *sup*E44 λ- *thi*-1 *gyr*A96 *rel*A1	Invitrogen
BL21	F- *omp*T *hsd*S_B_ (*r*_B_ *^−^m*_B_ *^−^*) *gal dcm rne1*31 (DE3)	Invitrogen
DB3.1	F- *gyr*A462 *end*A1 *gln*V44 Δ(sr1-*rec*A) *mcr*B *mrr hsd*S20(r_B_^−^, m_B_^−^) *ara*14 *gal*K2 *lac*Y1 *pro*A2 *rps*L20(Sm^r^) *xyl*5	Invitrogen
XL1-Blue	*recA1 endA1 gyrA96 thi-1 hsdR17* (r_k_^−^, m_k_^−^) *supE44 relA1 lac* (F^−^ *proAB lacI* ^q^ *Z* Δ*M15* Tn*10* (tet^r^))	Stratagene
Plasmids		
pDONR221	Entry vector, Km^r^	Invitrogen
pDEST17	Destination expression vector of the Gateway system (His-tag), Ap^r^	Invitrogen
pDEST24	Destination expression vector of the Gateway system (GST), GST donor, Ap^r^	Invitrogen
pTP145	Phage display vector, Cm^r^	37
pKRP13	Spectinomycin resistance gene donor, Sp^r^	53
pTP245	Phage display vector derived from pTP145, containing the *att*R1-Cm^r^-*ccd*B-*att*R2 sequence, Ap^r^	This study
pTP247	Expression vector derived from pDEST17, containing a stop codon at the position 1846, Ap^r^	This study
pTP248	Expression vector derived from pDEST17, containing both His- and GST tags, Ap^r^	This study
pTP249	Expression vector derived from pDEST17, containing a DNA fragment encoding a *pel*B signal sequence, Ap^r^	This study
pTP262	Phage display vector derived from pTP145, containing the *att*R1-Spe^r^-*ccd*B-*att*R2 sequence, Cm^r^, Sp^r^	This study
Primers		
*att*B1 forward	GGGGACAAGTTTGTACAAAAAAGCAGGCTTC-ORF specific bps^1^	Invitrogen
*att*B2 reverse	GGGGACCACTTTGTACAAGAAAGCTGGGTN-ORF specific bps^2^	Invitrogen
DONRF-2	GTTCCCTACTCTCGCGTTAACG	This study
DEST17-STOP	CAGCTTTCTTGTACAAAGTGGTTTGATTCGAGG	This study
PELB-F	TATGAAATACCTGCTGCCGACCGCTGCTGCTGGTCTGCTGCTCCTCGCTGCCCAGCCGGCGATGGCCCA	This study
PELB-R	ACTTTATGGACGACGGCTGGCGACGACGACCAGACGACGAGGAGCGACGGGTCGGCCGCTACCGGGTAT	This study
DEST-XHOI	GGGCTCGAGCTCTCGAATCAACAAGTTTGTACAAAAAAGCTGAACGAGAAACGTAAAATG	This study
DEST-XBAI	GGCGTCTAGACAACCACTTTGTACAAGAAAGCTGAACGAGAAACGTAAAATGATATAAATATC	This study
NPAP-F	TTGCGAGGCTCAGTTTTATCAC	This study
NPAP-R	CCAGAATTGGCGTAAAACATCA	This study

1, The 25 bp *att*B1site is underlined.

2, The 25 bp *att*B2 site is underlined, N represents A, G, C, or T.

### Primer Design

The predicted *S. oneidensis* MR-1 open reading frames (ORFs) based on the annotation by TIGR (www.tigr.org) were used for primer design. Signal peptides were determined using Signal-P prediction (http://www.cbs.dtu.dk/services/SignalP/) and the corresponding encoding DNA sequences were removed prior to primer design. Further analysis using PRIMEGENS was performed to identify repetitive ORFs, for which one pair of primers was designed for a group [52]. In total, 4526 unique pairs of primers were designed for *S. oneidensis*. The general guidelines for design were from manufacturer's recommendations (Invitrogen). The *att*B1 and *att*B2 sequences, listed in Table 2, were included at the 5′ ends of each of the ORF specific primer pairs. To maintain the proper reading frame between the *att*B1 and *att*B2 regions and the ORF, additional nucleotides (TC) and N (A, G, C, or T) were added to the 3′ ends of the *att*B1 and *att*B2 sequences, respectively, before the ORF-specific nucleotides (Table 2). As a result, one of four amino acids (Tyr, His, Asn, or Asp) will be inserted as the first amino acid C-terminal to the ORF as part of the *att* fusion region when the protein is translated from a destination vector. The ORF-specific region of the forward primers began with the start codon and the reverse primers began with the final nucleotide before the stop codon. The natural stop codon for each ORF was not included in the reverse primers. The annealing temperature of these primers was kept in the range of from 54 to 65°C by adjusting the length of primers (18–28 bp). The primers, given in Table S1, were synthesized by MWG (mwg-biotech.com) in 96-well format.

### PCR amplification of the ORFs

Amplification of the ORFs was performed in a 96-well format using in a GeneAmp PCR System 9700 thermal cycler (Applied Biosystems). The first attempt was made in a volume of 25 µl, with 3.125 U of *Pfu* Turbo polymerase, 1.5 X final concentration of a 10X cloned *Pfu* DNA polymerase buffer, 500 µM of each dNTP, 0.5 µM of each of Forward and Reverse primers, and 100 ng of genomic DNA based on the manufacturer's recommendation (Stratagene). Thermocycling was carried out using an initial denaturation step at 94°C for 2 min, followed by 30 cycles of denaturation at 94°C for 30 seconds, annealing at 55°C for 30 seconds, and elongation at 72°C for 2 min. After a final incubation at 72°C for 7 minutes, the reaction was held at 4°C. For longer ORFs (>2 kb), elongation time at 72°C was proportionally extended. PCR products were examined on a 1% agarose gel to confirm the size of the amplicons. For ORFs that failed to display the correct size of PCR product, at least three attempts were made with modified amplification conditions based on the annealing temperature of the corresponding primers. For ORFs that exhibited multiple bands including one of the correct size, gel purification of the correct size products was performed using QIAquick Gel Extraction Kit (Qiagen).

### BP recombinational cloning

PCR products of correct size were cloned into pDONR221 using the BP reaction kit (Invitrogen) following the protocol recommended by the manufacturer. Chemically competent *E.coli* DH5α cells (Invitrogen) were transformed with 2 µl of the BP reaction, spread onto LB-kanamycin plates, and incubated overnight. Kanamycin resistant *E.coli* DH5α transformants were screened using colony PCR with universal vector primers M13 Forward and Reverse. Visualization of a PCR product corresponding to the size of the ORF indicated the insertion of the ORF into the vector.

### Plasmid DNA isolation and DNA sequencing

Entry plasmid DNA was isolated for each clone using the QIAprep Spin Miniprep or QIAprep 96 Turbo Miniprep kits (Qiagen). Plasmid DNA was sequenced using Big Dye chemistry (Applied Biosystems) with the M13 Forward and M13 Reverse primers available from Invitrogen and/or with an additional forward primer DONRF-2 (Table 2). Sequence reads were compared to the *S. oneidensis* whole genome sequence or the pDEST17 plasmid sequence using BLASTn and BLASTx (NCBI, NLM). Discrepancies were verified by manual examination of the sequence traces. Custom oligonucleotides were purchased from Integrated DNA Technologies (idtdna.com).

### Plasmid constructions

a) Construction of pTP247

The stop codon of the destination vector pDEST17 (Invitrogen) is not in-frame with the upstream gene sequence when conversions are made using the pDONR221 vector. This results in the addition of 22 extra amino acids to the converted gene product. The position of the stop codon in pDEST17 was changed by site-directed mutagenesis using primer DEST17-STOP (Table 2). Compared with pDEST17, the new plasmid, named pTP247, contains a stop codon (TGA) at position 1846.

b) Construction of pTP248

To create a His/GST double tagged destination vector, a *Sal*I–*Nhe*I fragment containing the GST gene was released from pDEST24 by restriction enzyme digestion, and then inserted into pDEST17 between the *Sal*I and *Nhe*I sites. The resulting plasmid, named pTP248, contains a His tag and a GST tag flanking the target gene at the N-terminal and C-terminal sites, respectively.

c) Construction of pTP249

To create a destination plasmid that can be used to express secreted or periplasmic proteins, a DNA fragment encoding a *pelB* signal sequence was created by annealing synthetic oligonucleotides (Table 2) and inserting the resulting DNA fragment into the *Nde*I restriction site of the pDEST17 plasmid. The resulting plasmid was named as pTP249.

d) Construction of pTP262

In order to construct a destination vector for M13 phage display, the *att*R1-Cm^r^-*ccdB*-*att*R2 region of the pDEST17 destination vector was amplified by PCR using primers DEST-XHOI and DEST-XBAI (Table 2).

The resulting product was digested with the *Xho*I and *Xba*I restriction enzymes and inserted into the pTP145 phage display vector digested with *Sal*I and *Xba*I [37]. The resulting plasmid, named pTP245, has the *att*R1 site fused adjacent to the *bla*TEM-1 signal sequence for protein secretion and the *att*R2 site fused adjacent to M13 gene III for phage display [37]. The pTP145 plasmid, however, contains a chloramphenicol resistance gene and therefore the Cm^r^ gene present in the *att*R cassette is not useful for selection. To insert a useful selectable marker, the Cm^r^ gene in the *att*R cassette was replaced with a spectinomycin resistance gene from the plasmid pKRP13 [53]. This was accomplished by creating *Hind*III restriction sites at the *Not*I and *Bgl*II sites of pTP245 which flank the Cm^r^ resistance gene in the *att*R1-*att*R2 cassette by oligonucleotide directed mutagenesis. The Cm^r^ resistance gene was then replaced by inserting the spectinomycin gene on 2.0 kbp *Hind*III fragment of pKRP13 [53]. The resulting plasmid, named pTP262, contains an *att*R1-Sp^r^-*ccdB*-*att*R2 cassette between the signal sequence of β-lactamase and M13 phage gene III (Fig. 3).

### Expression of two-component regulatory proteins

The experiments to determine protein expression levels of two-component response regulatory proteins in *E. coli* were initiated by converting the relevant pDONR-ORF plasmids to the pTP247 His-tag destination plasmid using a Clonase reaction according to manufacturer instructions (Invitrogen). The resulting pTP247-ORF plasmids were introduced into *E. coli* BL21(DE3)* for protein expression experiments. *E. coli* BL21(DE3)* strains containing the expression plasmids were inoculated into 15 ml of LB with ampicillin and grown to an OD_600_≈1.0, at which point protein expression was induced by the addition of IPTG to a concentration of 1 mM and the cultures were grown overnight. After overnight growth, 1 ml of each induced culture was centrifuged at 10K rpm for 1 min. to pellet cells. The supernatant was discarded and the cell pellet was resuspended in 300 µl of bacterial protein extraction reagent (B-PER, Pierce) and 6 µl of 10 mg/ml lysozyme was added. The cell suspension was frozen at −80°C and then thawed at room temperature and centrifuged at 13K rpm for 10 min. The precipitated proteins in the pellet were resuspended in 30 µl SDS gel loading buffer and 20 µl of soluble supernatant was mixed with 30 µl of SDS gel loading buffer. The protein preparations were then heated to 95°C and fractionated by SDS-PAGE. The SDS-PAGE gels were stained with Coomassie blue and the level of protein expression was judged by the presence or absence of a band of the expected size on the gel.

### Purification of NarP protein and use in gel shift assays

For purification of NarP, the pTP247-*narP* was introduced into *E. coli* BL21(DE3)*. This strain was grown in 1 liter of LB with ampicillin to an OD_600_ of 1.0 and induced with 1 mM IPTG and grown overnight. The cells were centrifuged and the pellet was resuspended in 50 ml of 1X Equilibration buffer (50 mM sodium phosphate buffer, pH 7.0, 300 mM NaCl). A protein lysate was made from the resuspended cells using a microfluidizer instrument. The lysate was centrifuged at 14K rpm for 20 min. to remove insoluble material. NarP was purified from the protein lysate by metal affinity chromatography using a TALON resin (Clontech) that had been equilibrated in 1X Equilibration buffer and protein was eluted with 50 mM sodium phosphate, 300 mM NaCl, and 150 mM immidazole. The purity of the eluted fractions was evaluated by SDS-PAGE coomassie-stained gels. Fractions containing >90% pure NarP were pooled and dialyzed against 50 mM HEPES buffer. The NarP protein was concentrated by ultrafiltration using an Amicon Ultra-15 column and the protein concentration was determined using a Bradford assay [54]. Electrophoretic mobility shift assays were performed for NarP with minor modifications as described for ArcA by Gao et al. [33]. The modification for the NarP experiments is that the shifted and unshifted DNA was visualized using SYBR Green staining rather than by labeling the DNA with ^33^P. The target *narP-nrfA* promoter DNA for the EMSA experiments is a 527 bp PCR product obtained using the primers NPAP-F and NPAP-R (Table 2). This region corresponds to nucleotide coordinates 4117504 to 4118031 in the *Shewanella oneidensis* MR1 genome sequence [1].

### Western blotting of *Shewanella* proteins expressed from pTP248

To examine expression levels of His/GST double tagged proteins, the *so0037* to *so0059* ORFs were converted from the pDONR221 plasmid to the pTP248 plasmid using LR clonase according to manufacturer instructions. The SO-orf-pTP248 clones were introduced into *E. coli* BL21(DE3)* by electroporation and individual cultures were grown in 10 ml of LB supplemented with ampicillin to an OD_600_ of approximately 0.6 and protein expression was induced by adding IPTG to a final concentration of 0.3 mM. The cultures were then grown overnight. The samples were centrifuged to pellet cells and the cells were resuspended in SDS-PAGE loading buffer. The amount of loading buffer used was adjusted to the OD_600_ of the cultures to maintain a constant OD_600_ to loading buffer ratio. A total of 15 µl of each sample was fractionated by SDS-PAGE and the proteins were transferred to a nitrocellulose filter. Western blotting was then performed as previously described [55] using a 1:5000 dilution of rabbit anti-GST polyclonal antibody purchased from Amersham.

### Phage display biopanning

Plasmid DNA from pDONR clones within the *so0337* to *so0516* region was prepared and the set of 80 plasmids was pooled. The pooled plasmids were then converted from the pDONR vector to the pTP262 destination vector in batch using Clonase recombination enzyme in 10 µl reaction volume. The reaction products were introduced into *E. coli* XL1-Blue by electroporation and the transformed cells were spread on agar plates containing chloramphenicol. Six colonies were picked and analyzed by restriction enzyme digests to ensure the conversion occurred efficiently and the library consisted of inserts into the pTP262 plasmid. Five of the six colonies analyzed contained an insert and the inserts were of different size. The colonies on the agar plates were then pooled to create a sub-library of *S. oneidensis* genes in pTP262.

Biopanning was performed by coating a microtiter ELISA well with 10 µg/ml of rabbit polyclonal anti-*E. coli* thioredoxin antibody (Sigma). After absorption, the wells were blocked with 3% dry milk, 0.05% Tween 20 in 1x TBS. Approximately 1×10^11^ phage were added to the well in 1x TBS and incubated to allow binding. After 10 cycles of washing with 1x TBS, bound phages were eluted using 0.2 M glycine, pH 2.2. The elution was neutralized with 1 M Tris pH 8.0 and the eluate was used to infect *E. coli* TG1. VCS M13 helper phage were added and the culture was grown overnight and phages were prepared as previously described [37]. This process was repeated three times to enrich for phage that displayed a protein that bound the anti-thioredoxin antibody. After the third round, the eluted phage were used to infect *E. coli* TG1 and the transfections were spread on LB agar plates containing chloramphenicol to isolate single colonies. Six colonies were picked and the phagemid DNA was isolated and the DNA sequence of the ORF region was determined by automated DNA sequence using Big Dye Terminator and an Applied Biosystems 3100 capillary DNA sequencer.

## Supporting Information

Table S1 S. oneidensis clone set ORF-specific primers.
(0.69 MB XLS)
